# Effects of *Klf4* and *c-Myc* Knockdown on Pluripotency Maintenance in Porcine Induced Pluripotent Stem Cell 

**DOI:** 10.22074/cellj.2018.4428

**Published:** 2017-11-04

**Authors:** Yu-Jing Liao, Yi-Shiou Chen, Ja-Xin Lee, Lih-Ren Chen, Jenn-Rong Yang

**Affiliations:** 1 Division of Physiology, Livestock Research Institute, Council of Agriculture, Executive Yuan, Tainan, Taiwan; 2 Department of Animal Science, National Chung Hsing University, Taichung, Taiwan; 3 Hsinchu Branch, Livestock Research Institute, Council of Agriculture, Executive Yuan, Hsinchu, Taiwan; 4 Institute of Biotechnology, National Cheng Kung University, Tainan, Taiwan; 5 Institute of Biotechnology, Southern Taiwan University, Tainan, Taiwan

**Keywords:** *c-Myc*, *Klf4*, Pluripotency, Short Hairpin RNA

## Abstract

**Objective:**

The importance of *Oct4* and *Sox2* in maintaining pluripotency and self-renewal is well-understood, but the functions of *Klf4* and *c-Myc* has not been fully investigated. In the present study, we attempted to determine the roles of Klf4 and c-Myc on pluripotency maintenance of porcine induced pluripotent stem (piPS) cells.

**Materials and Methods:**

In this experimental study, we performed short hairpin RNA (shRNA) to knock down the *Klf4* and *c-Myc* functions of piPS cells and examined pluripotency markers and teratoma formation to evaluate piPS cell pluripotency. The shRNA-Klf4 and shRNA-c-Myc vectors containing a reporter gene, TagFP635, were transfected into piPS cells by lentivirus infection. The piPS cells fully expressing infrared fluorescence were selected to confirm gene knockdown of *Klf4* and *c-Myc* reverse transcription-polymerase chain reaction (RT-PCR). Next, for pluripotency evaluation, expression of pluripotency markers was detected by immunocytochemical staining, and capability of teratoma formation was investigated by piPS cell transplantation into nonobese diabetic-severe combined immunodeficiency (NOD-SCID) mice.

**Results:**

Our findings indicated that *Klf4* and *c-Myc* functions of piPS cells were knocked down by shRNA transfection, and knockdown of *Klf4* and *c-Myc* functions impaired expression of pluripotency markers such as Oct4, AP, SSEA-3, SSEA-4, TRA-1-6, and TRA-1-81. Furthermore, piPS cells without *Klf4* and* c-Myc* expression failed to form teratomas.

**Conclusion:**

The pluripotency of piPS cells are crucially dependent upon *Klf4* and *c-Myc* expression. These findings, suggesting potential mechanisms of *Klf4* and *c-Myc* contribution to piPS cell formation, have important implications for application, regulation, and tumorigenesis of piPS cells.

## Introduction

Self-renewal and pluripotency of embryonic stem (ES) cells are regulated by many transcription factors. Among them, *Oct4, Sox2*, and *Nanog* are well-known and thought to be the master regulators of ES cell pluripotency (1, 2). By inducing expression of *Oct3/4, Sox2, Klf4,* and *c-Myc,* induced pluripotent stem (iPS) cells are first generated from mouse embryonic and adult fibroblasts and resemble the property of ES cells. These four factors use distinct mechanisms to maintain the pluripotency of iPS cells. The importance of *Oct4* and *Sox2* in ES cell pluripotency maintenance and selfrenewal is well-understood, but the functions of *Klf4* and *c-Myc* have not been fully investigated (3). *Oct4* is essential for regulation of early embryonic differentiation, maintenance of pluripotency (4, 5), preventing ES cell differentiation, and sustaining ES cell self-renewal (5). *Sox2* collaborates with *Oct4* to regulate gene expression (6, 7). *Klf4* is expressed in various tissues and involves proliferation, terminal differentiation, and apoptosis (8). In addition, *Klf4* can either activate or repress transcription and can act as either an oncogene or a tumor suppressor (9, 10). These results suggest that *Klf4* might be an important regulator of ES cell self-renewal and pluripotency. *c-Myc* has been reported as an enhancer for reprogramming but might be redundant (11, 12). However, without *c-Myc*, the efficiency of iPS cell production is dramatically reduced, suggesting an important role for maintenance of pluripotency (11). Genomics studies have suggested that *c-Myc* acts as a repressor of fibroblastspecific gene, and that might elucidate its importance in the early reprogramming process in iPS cells (13).

Teratoma formation analysis is a well-known protocol for determination of *in vivo* differentiation capability of human and murine ES cells (14, 15). However, porcine ES (pES) cells hardly develop teratomas (16). In fact, teratomas can be formed from pES cells derived from late stage of blastocysts (10-11 days), but not early stage of blastocysts (5-6 days) (16- 18). As our previous study, pES cells established from day 7 blastocysts are also unable to induce teratoma formation (19). On the other hand, when porcine induced pluripotent stem (piPS) cells are transplanted into NOD-SCID mice, the development of teratomas is efficient (20-22). The result of teratoma formation between pES and piPS cells is still elusive. Thus, for clinical application, teratoma formation should be concerned. RNA interference (RNAi) is a powerful technique to study gene function. Small interfering RNAs (siRNAs) and microRNAs (miRNAs) are short noncoding RNA duplexes with important roles in gene regulation (23, 24), having distinct mechanisms, that target messenger RNAs (mRNAs) to silence gene expression (23). Unlike siRNAs which are chemically synthesized, short hairpin RNAs (shRNAs) are vector based. shRNAs are stem-loop RNAs and expresse in the nucleus. Subsequently, they are transported to the cytoplasm for further processing in the same manner as siRNAs (25). In the present study, we compare teratoma formation between pES and piPS cells, and use shRNA to knock down the expression of *Klf4* and *c-Myc* of piPS cells. The expression of pluripotency markers and the capability of teratoma formation were examined to investigate the importance for pluripotency maintenance of piPS cells.

## Materials and Methods

### *In vitro* culture of porcine embryonic stem cells and porcine induced pluripotent stem cells

The piPS cells used in this experimental study were generated from porcine ear fibroblasts transfected with human *OCT4, SOX2, KLF4*, and *c-MYC* genes constructed in lentivirus vectors (TLC-TRE-iPS-II, Tseng Hsiang Life Science LTD, Taipei, Taiwan) and maintained in ES cell culture medium as our previous study (22). The pES cells were established from the inner cell mass (ICM) in preimplantation blastocysts of the Taiwan Livestock Research Institute Black Pig No. 1, as in our previous study (19). Both types of porcine pluripotent stem cells were propagated on the feeder layer of mitomycin C (Sigma-Aldrich, St. Louis, MO, USA)-inactivated STO cells (mouse embryonic fibroblasts, CRL-1503, USA) in 0.1% gelatin-coated Multidish 4 Wells® (Nunc 176740, Rosk ilde, Denmark) and cultured at 37˚C under an atmosphere of 5% CO_2_ in air. For passaging piPS and pES cells, pluripotent colonies were dissected into small clusters by fine pulled Pasture pipette and transferred to the new feeder layer (19, 22, 26-28).

### The short hairpin RNA transfection

Custom shRNA-Klf4 and shRNA-c-Myc with the nucleotide sequences of GATGGCTGTGGGTGGAAATTT and GAGGCGAGAACAGTTGAAACT, respectively, were constructed by Sigma-Aldrich. To enhance the efficiency of lentivirus infection, STO cells were removed by sterilized pipette tips before infection and 2-4 μL of hexadimethrine bromide (polybrene) was added. Multiplicity of infection (MOI) is the number of lentiviral particles per cell in the transduction. Because piPS cells were in form of extreme aggregation of cells, a precise MOI is hard to be calculated, therefore, a range of MOI (9-18) was tested. The vehicle, shRNA-Klf4, and shRNA-c-Myc vectors containing a reporter gene (TagFP635) were introduced into piPS cells by lentivirus infection for 20 hours according to the manufacturing protocol (Sigma-Aldrich, St. Louis, MO, USA). The vehicle vector was used as a control to test the condition of MOI and polybrene for lentivirus infection. After infection for 20 hours, the infection medium was removed, and piPS cells were maintained in ES cell culture medium to monitor the expression of infrared fluorescence. Full signal of infrared fluorescence in piPS cells indicated successful transfection, and the cells were picked up by fine pulled Pasture pipette and maintained on the new feeder layers. The image of transfected cells was observed by the inverted microscopy (DM IRB, Leica, Wetzlar, Germany) and captured by monochrome microscope camera (DS-Qi2, Nikon, Melville, NY, USA).

### Gene expression of *Klf4* and *c-Myc*

To verify the knockdown of *Klf4* and *c-Myc* after shRNA transfection, total RNA of transfected piPS cells was isolated using PureLink™ RNA mini kit (Ambion, Grand Island, NY, USA) and reverse-transcribed into cDNA with transcriptor first strand cDNA synthesis kit (Roche, Indianapolis, IN, USA). The cDNA was subjected to reverse transcription-polymerase chain reaction (RT-PCR) with following conditions: initial denaturation for 5 minutes at 94˚C, 32 cycles of denaturation for 30 seconds at 94˚C, annealing for 30 seconds at 60˚C, and elongation for 1 minute at 72˚C, and post-elongation for 3 minutes at 72˚C. *β-actin* was as an endogenous control. The relative expression of *Klf4* and *c-Myc* was measured by Image J software. The primers were listed in Table 1.

**Table 1:** The primer lists for reverse-transcription polymerase chain reaction (RT-PCR)


Gene	Primer sequence (5ˊ-3ˊ)	Length (bp)

*Klf4*	F: GCGGAGGAACTGCTAAG	423
	R: GCACTTCTGGCACTGGA	
*c-Myc*	F: TCGGACTCTCTGCTCTCCTC	274
	R: CTGCATAATTGTGCTGGTGC	
*β-actin*	F: TGGATGACGATATCGCTGCGC	598
	R: AAGCTGTAGCCACGCTCGGTC	


### Characterization of the pluripotency markers

For immunocytochemical staining, piPS cells were fixed in 10% (v/v) neutral buffered formalin and stained with specific antibodies. For 3-Amino-9-ethylcarbazole (AEC) staining, piPS cells were permeabilized with 0.3% (v/v) Triton X-100 for 10 minutes, fixed by formalin, and then incubated with 0.3% H_2_O_2_ for 5 minutes. Finally, the cells were incubated with blocking solution [5% (v/v) fetal bovine serum (FBS) in phosphate buffered saline (PBS) containing 0.1% (v/v) Tween-20] for 2 hours at room temperature. The cells were incubated with primary antibody diluted with blocking solution at 4˚C overnight. On the next morning, after incubated with horseradish peroxidase-conjugated secondary antibody diluted with blocking solution for 2 hours at room temperature, the cells were stained by AEC kit (Sigma- Aldrich, St. Louis, MO, USA). Primary antibodies used in the present study included octamer-binding transcription factor 4 (Oct4, Millipore Cat. #AB3209, Temecula, CA, USA), alkaline phosphatase (AP, Millipore Cat. #MAB4349), stage specific embryonic antigen-3 (SSEA-3, Millipore Cat. #MAB4303), stage specific embryonic antigen-4 (SSEA- 4, Millipore Cat. #MAB4304), tumor related antigen-1-60 (TRA-1-60, Millipore Cat. #MAB4360), and tumor related antigen-1-81 (TRA-1-81, Millipore Cat. #MAB4381). The secondary antibodies for AEC staining were horseradish peroxidase conjugated AffiniPure goat anti-rabbit IgG (for Oct4 staining, Jackson ImmunoResearch Cat #111-032-003), rabbit anti-mouse IgG (for AP and SSEA-4 staining, Jackson ImmunoResearch Cat #315-035-003), rabbit anti-rat IgM (for SSEA-3 staining, Jackson ImmunoResearch Cat #312-035- 020), and rabbit anti-mouse IgG + IgM (H + L) (for TRA- 1-60 and TRA-1-81 staining, Jackson ImmunoResearch Cat #315-035-044). The image of stained cells was observed by the inverted microscopy (TE300, Nikon) and captured by digital camera (D700, Nikon).

### Teratoma formation

For teratoma formation analysis, sixteen NOD-SCID mice (Bio-LASCO, Taiwan) at 8 weeks of age were used for cell transplantation. We designed two experiments to investigate the teratoma formation. In experiment 1, the purpose was to compare teratoma formation efficiency between pES and piPS cells. The suspension of 1×10^6^ of pES and piPS cells in 100 μL of PBS was subcutaneously injected into the right and left dorsal flanks of the same NOD-SCID mice, respectively (n=7). In experiment 2, the purpose was to examine teratoma formation capability between piPS cells and piPS cells without *Klf4* and *c-Myc* expression. The suspension of 1×10^6^ of piPS and piPS cells without *Klf4* and *c-Myc* expression in 100 of μL PBS was subcutaneously injected into the left dorsal flanks of NOD-SCID mice (n=3, each group, n=9, total). The length, width, and height of teratomas were measured every two weeks during the eight-week experimental period

### Statistical analysis

Data were analyzed by analysis of variance using the General Linear Model (GLM) procedure and Duncan’s multiple range test of SAS (SAS Enterprise Guide 4.1. SAS Institute Inc., Cary, North Carolina, USA). The significant difference was determined as the P<0.05.

### Ethical considerations

All animal experiments in this study and the procedures for animal handling and treatments were approved by the Livestock Research Institutional Animal Care and Use Committee (no. 104-33).

## Results

### The porcine embryonic stem cells failed to induce teratoma formation

**Fig.1:**
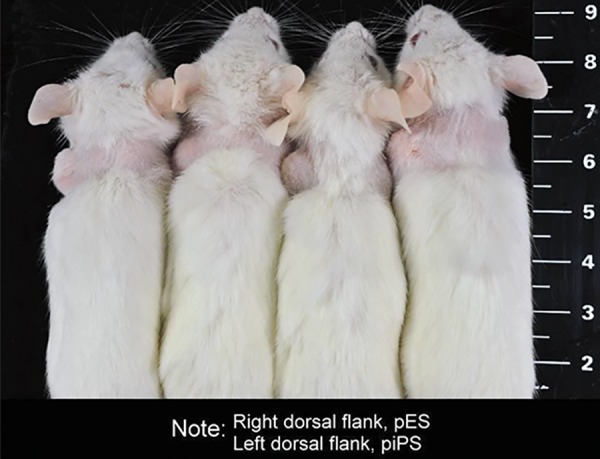
The porcine embryonic stem (pES) cells are unable to develop teratoma. pES cells in the right dorsal flank failed to induce teratoma formation, but porcine induced pluripotent stem (piPS) cells in the left dorsal flank efficiently developed into teratomas.

To compare teratoma formation capability, pES and piPS cells were subcutaneously transplanted into the right and left dorsal flanks of the same NOD-SCID mice, respectively. Eight weeks after transplantation, teratoma formation induced by piPS cell transplantation was obvious in the left dorsal flank of mice while the right dorsal flank, which had been injected with pES cells, did not show any teratomas (Fig .1).

### Knockdown of *Klf4* and *c-Myc* disturbed the morphology

**Fig.2:**
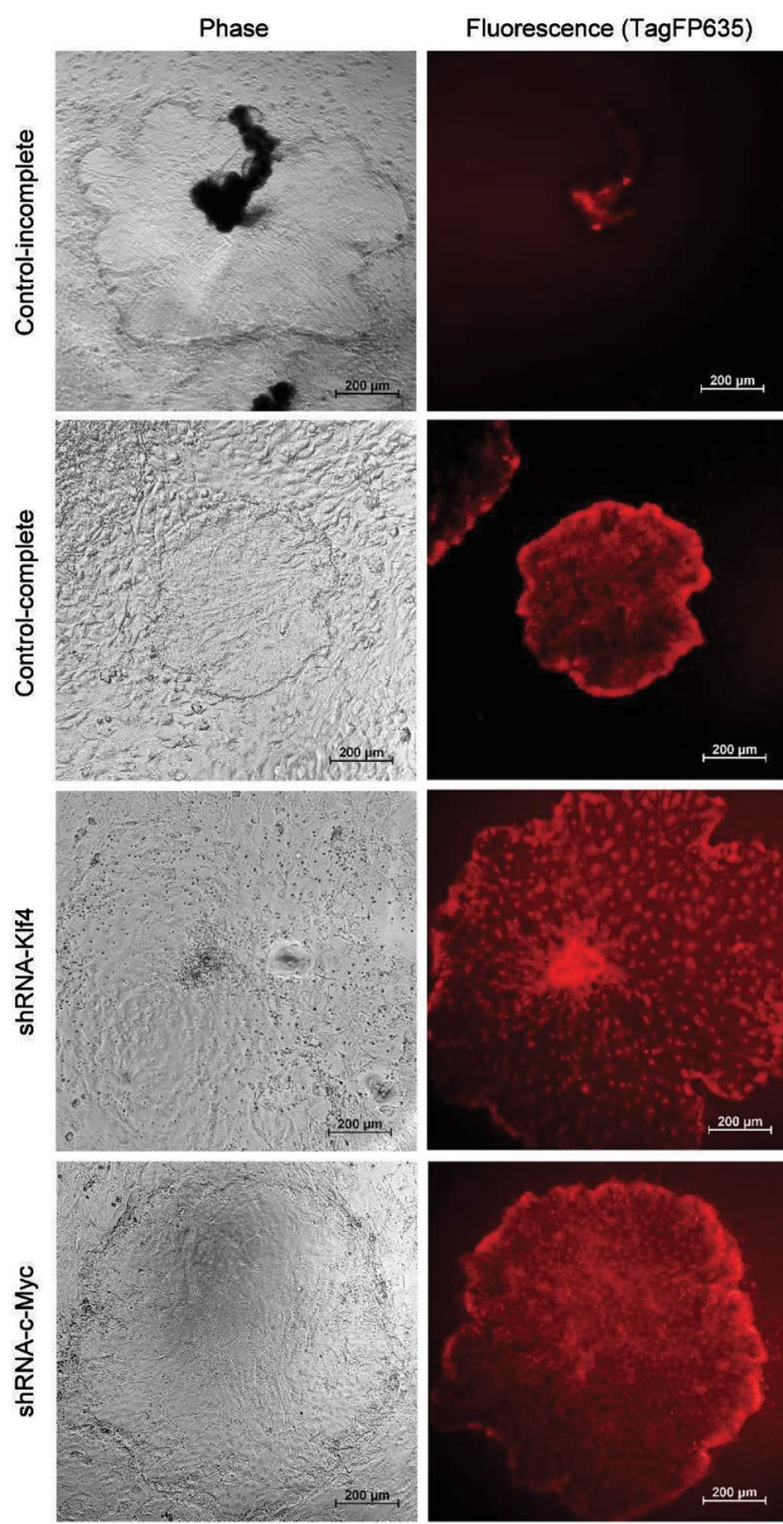
The expression of infrared fluorescence is an indicator of successful transfection. The expression of infrared fluorescence was incomplete at low MOI, but enhanced at high multiplicity of infection (MOI). After knockdown of *Klf4* and *c-Myc*, porcine induced pluripotent stem (piPS) cells showed loose morphology and scattered nuclei.

**Fig.3:**
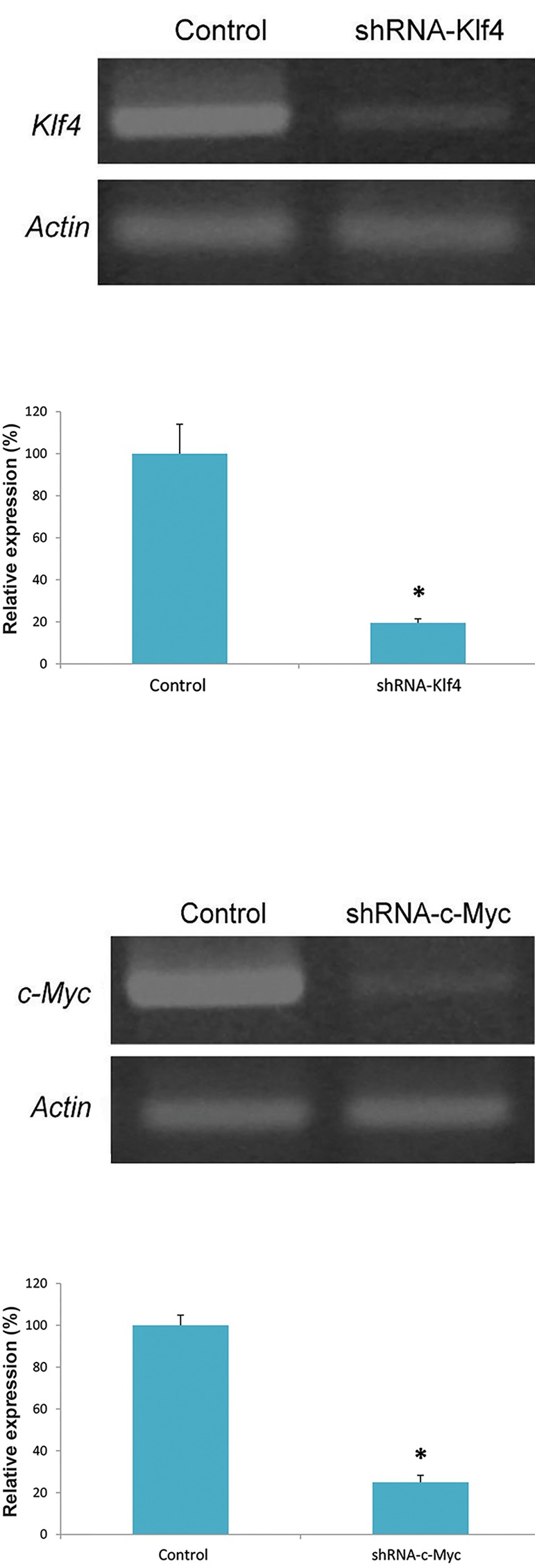
The shRNA knockdown *Klf4* and *c-Myc* expression. The expression of *Klf4* and *c-Myc* was inhibited by 80 and 75%, respectively. *; P<0.05 (Duncan’s multiple range test).

of porcine induced pluripotent stem cells To optimize the best condition for lentivirus infection, various MOI and concentrations of polybrene were tested, and the high intensity of infrared fluorescence in infected cells was used as an indicator for successful infection. At first, we used lentivurus containing vehicle vectors as a control to infect piPS cells by using the condition of MOI of 9 with 2 or 4 μL of polybrene. Ideally, the infrared fluorescence in each cell will express one week after transfection. However, the infrared fluorescence only expressed in the middle of each colony, where piPS cells aggregated and was indiscernible when piPS cells proliferated outwards (Fig .2). We assumed that the poor intensity of infrared fluorescence was due to low MOI. Thus, a high MOI of 18 with 2 μL of polybrene was tested again. Indeed, a high MOI enhanced the infection efficiency, and piPS cells maintained high infrared fluorescence intensity during their proliferation (Fig .2). Thereafter, shRNA-Klf4 and shRNA-c-Myc vectors were transfected into piPS cells under the same condition. One week after lentivirus infection, the transfected piPS cells expressed infrared fluorescence and were transferred to the new feeder layers by fine pulled Pasture pipette. In control groups, piPS cells maintained the compact and ES-like colony morphology. However, after shRNA transfection, the morphology of each piPS cell in the colony was distinct and showed discernible boundary to other cells. In addition, piPS cells transfected with shRNA-Klf4 and shRNA-c-Myc showed scattered nuclei under the infrared fluorescence imaging (Fig .2). RT-PCR results revealed that shRNA-Klf4 and shRNA-c-Myc had knocked down the expression of *Klf4* and *c-Myc* by 80 and 75%, respectively (Fig .3).

### The porcine induced pluripotent stem cells without

#### *Klf4* and *c-Myc* expression lost pluripotency

**Fig.4:**
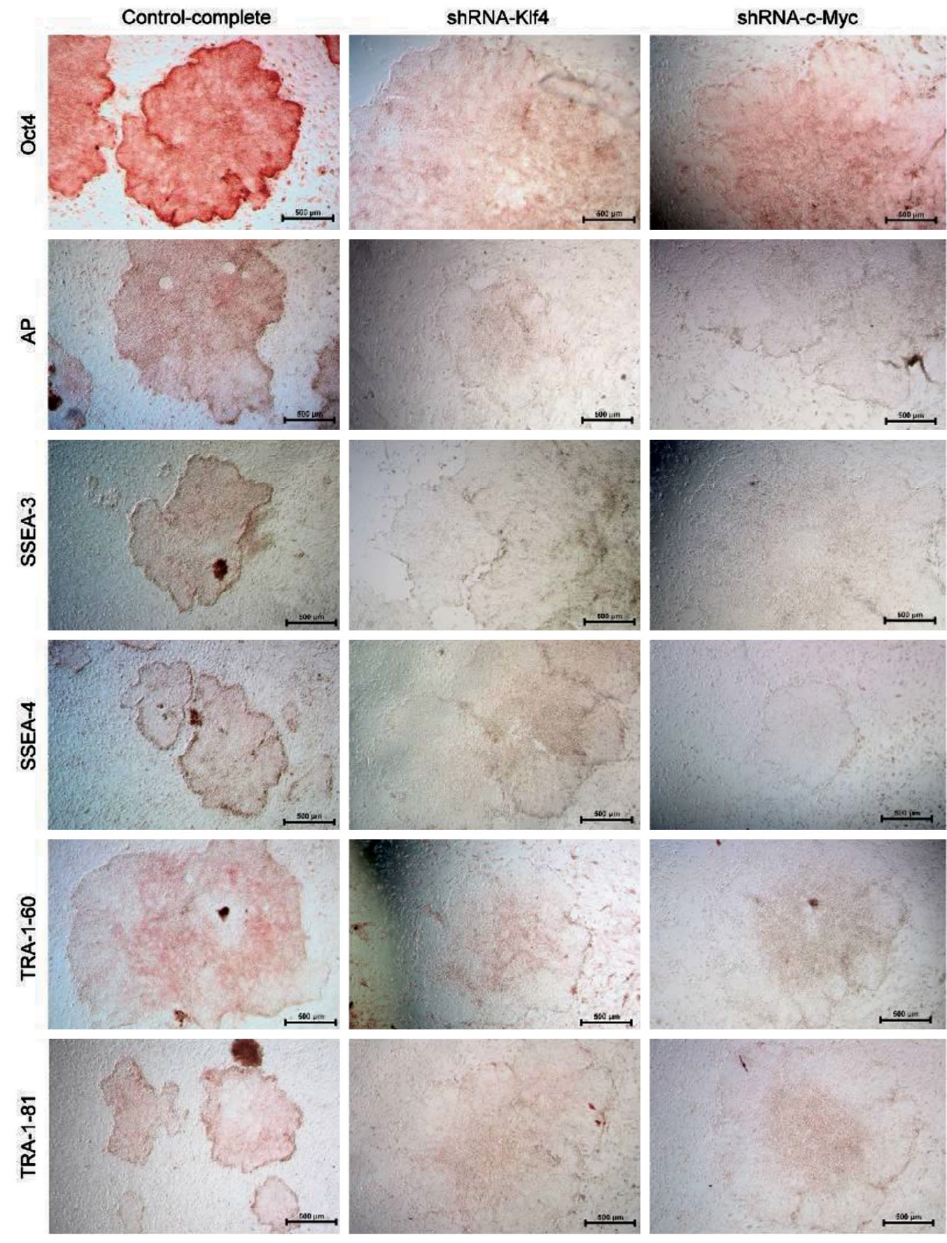
Knockdown of *Klf4* and *c-Myc* in porcine induced pluripotent stem (piPS) cells attenuate the expression of pluripotency markers. P<0.05 (Duncan’s multiple range test).

For determination of pluripotency of piPS cells, responding to shRNA-Klf4 and shRNA-c-Myc transfection, AEC staining and antibodies against pluripotency markers Oct4, AP, SSEA-3, SSEA-4, TRA-1-60, and TRA-1-81 were performed. All the pluripotency markers were positively detected in control groups, and the expression of Oct4 was the highest. However, after knockdown of *Klf4* and *c-Myc*, the expression of Oct4 in piPS cells was quite low and other pluripotency markers were almost undetectable (Fig .4).

### Knockdown of Klf4 and c-Myc inhibited teratoma formation of porcine induced pluripotent stem cells

**Fig.5:**
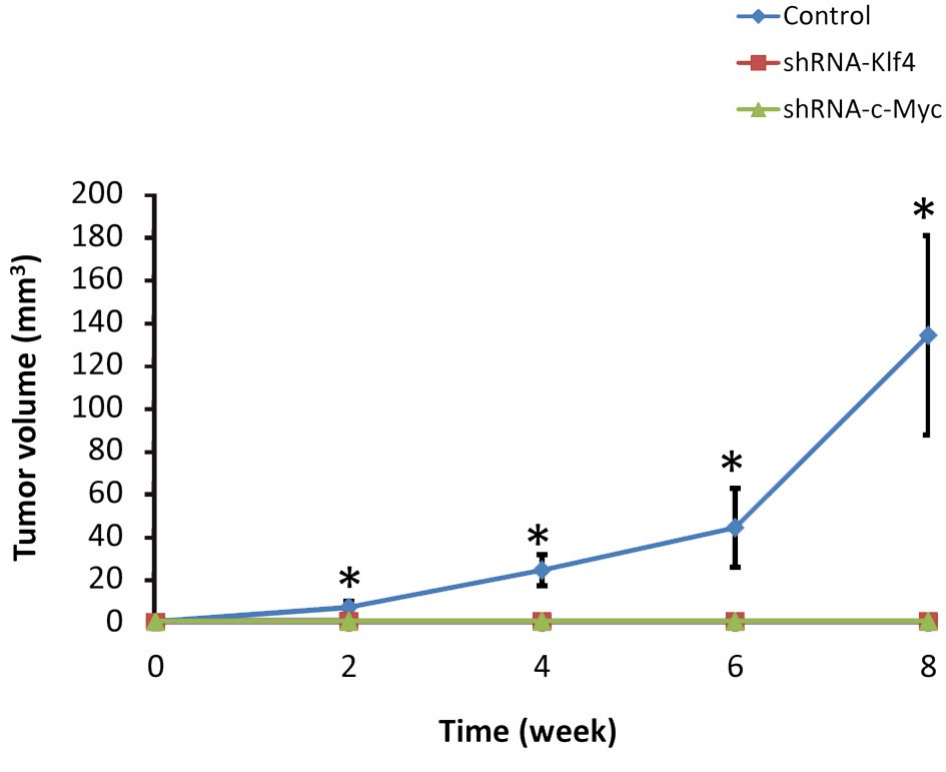
The porcine induced pluripotent stem (piPS) cells without *Klf4* and *c-Myc* function fail to induce teratoma formation. The teratomas were examined every two weeks during the eight-week experimental period. *; P<0.05 (Duncan’s multiple range test).

To determinate the influence of *Klf4* and *c-Myc* knockdown on teratoma formation, control piPS cells and their shRNA-Klf4 and shRNA-c-Myc transfected counterparts were subcutaneously injected into the left dorsal flank of NOD-SCID mice. Two weeks after transplantation, teratomas induced by control piPS were developed to 23.90 ± 7.26 mm3 in size and reached 133.63 ± 46.60 mm3 by eight weeks after transplantation. Contrarily, no teratoma formation was found in the NOD-SCID mice after transplantation of shRNA-Klf4 and shRNA-c-Myc transfected piPS cells during the eight-week experimental period (P<0.05) (Fig .5).

## Discussion

Although ES and iPS cells are pluripotent cells, one of the most important questions is their actual similarity (29). Previous studies showed that teratoma formation is hardly induced by pES cells (16), but the development of teratomas derived from piPS cells is efficient (20-22). We compared teratoma formation capability between pES and piPS cells by ectopic transplantation into NOD-SCID mice, and only piPS cells induced teratoma formation. This result reconfirms our previous studies (19), but the reasons are still unrevealed. Some unknown mechanisms contribute to the pluripotency maintenance and teratoma formation of piPS.

In the present study, we demonstrated the important roles of *Klf4* and *c-Myc* of piPS cells in preventing differentiation and in maintenance of self-renewal and pluripotency. *Klf4* is highly expressed in undifferentiated ES cells and also prevents ES cell differentiation through regulating *Nanog* gene expression (3). However, the expression dramatically diminishes during differentiation (30), and re-expression of *Klf4* reverts the pluripotent state (31). Knockdown of *Klf4* expression through Klf4 shRNA also reveals its importance in maintenance of pluripotency as well as self-renewal of ES cells. *Klf4* shRNA is stably expressed in ES cells through lentiviral infection, and knockdown of *Klf4* induces ES cell differentiation (3).

c- and N-Myc are essential for maintenance of ES cell pluripotency and self-renewal. Knockout of both *c- *and *N-Myc* promotes cell cycle arrest and apoptosis and disrupts ES cell pluripotency and self-renewal. Furthermore, loss of *c- *and *N-Myc* also induces ES cells to differentiate into ectoderm, mesoderm, and endoderm (32). *c- *and *N-Myc* are the key factors for early embryogenesis. Without them, embryos are hard to develop and exhibit various defects. In addition, knockdown of *c-Myc* inhibits tumor formation of nasopharyngeal carcinoma 5-8F cells in nude mice (33). Therefore, *Myc* genes are critical to maintain the pluripotency and self-renewal of ES cells, and this result shows important implications for iPS cells (32). In the present study, knockdown of *Klf4* and *c-Myc* function by shRNA disturbed morphology of piPS cells, suggesting the important roles of *Klf4* and *c-Myc* for the maintenance of piPS cell pluripotency and self-renewal.

The capability of teratoma formation is a standard procedure to examine the pluripotency of ES or iPS cells, but this capability will be completely lost after differentiation. The transplanted cells contaminated with undifferentiated pluripotent stem cells (34, 35) would induce teratoma formation (15, 36). Indeed, only 100 of human ES cells can generate teratomas, although the efficiency is low (37). ES and iPS cells can differentiate into specific cells and have high potential to ameliorate specific diseases. Therefore, the possibility of teratoma formation should be seriously considered for clinical application of stem cells. To avoid teratoma formation, the undifferentiated pluripotent stem cells should be removed from differentiated cells before transplantation (38). Many techniques have been devoted to remove undifferentiated cells, such flow cytometry (39), specific antibodies (40), tumor inhibitors (41), and some synthetic small molecules (42).

## Conclusion

Our findings indicate that pluripotency of piPS cells are crucially dependent upon *Klf4* and c-Myc expression. Knockdown of *Klf4* and *c-Myc* functions in piPS cells disturbs morphology, induces differentiation, and inhibits teratoma formation. These findings might have important implications for application, regulation, and tumorigenesis of piPS cells, and suggest potential mechanisms of *Klf4* and *c-Myc* in contributing to piPS cell formation.
